# Beyond silos: transdisciplinary collaboration for allied health clinicians providing stroke primary care

**DOI:** 10.3389/fmed.2026.1765278

**Published:** 2026-04-15

**Authors:** Aleysha K. Martin, Kerrie Evans, E-Liisa Laakso

**Affiliations:** 1 Allied Health, Mater Research Institute - University of Queensland, Brisbane, QLD, Australia; 2 Faculty of Medicine and Health, The University of Sydney, Sydney, NSW, Australia; 3 Healthia Limited, Brisbane, QLD, Australia

**Keywords:** integrated care, interprofessional, models of care, multidisciplinary, scope of practice

## Abstract

Integrating healthcare through team collaboration has many benefits, including more efficient, cost-effective services and improved client outcomes. However, in primary care, allied health professionals treating people with stroke often work in parallel, discipline-specific silos, which limits communication and leads to fragmented and/or duplicated care. Strengthening patient care after stroke requires moving beyond multidisciplinary collaboration toward transdisciplinary collaboration. Transdisciplinary collaboration is a form of skill-sharing, where one professional completes additional competency training to safely work beyond their usual scope of practice. In this perspectives paper, we present the view that transdisciplinary allied health collaboration has a role in primary care, using stroke as our case example. We explore the potential benefits and feasibility of transdisciplinary collaboration and provide clinical examples and insights to illustrate the concepts. We propose five considerations when embarking on transdisciplinary collaboration: (1) healthcare settings and jurisdiction, (2) consumer needs, (3) workforce availability, (4) clinician attributes and skills, and (5) team collaboration rules. This paper challenges allied health professionals, leaders and managers working in primary care, as well as policy makers, to review current practice and explore avenues for transdisciplinary collaboration.

## 1 Introduction

In this paper, we explore transdisciplinary collaboration in primary care. Transdisciplinary collaboration has the potential to better integrate primary care. We explore this concept within a novel context – allied health clinicians providing care after stroke in primary care settings. There is a lack of transdisciplinary literature in primary care. Therefore, we draw on the related field of integrated care and combine this with the operational and clinical knowledge and experience from professionals currently working in primary care. We define integrated care as healthcare professionals from multiple disciplines working together to address care needs (1, 2). When care is provided by an integrated team, there is less fragmentation of care, improved interprofessional communication and patients benefit from more comprehensive care, achieving better outcomes (1, 3). For allied health professionals working in primary care, the most frequently described model of integrated care is multidisciplinary collaboration (4). While we recognize that there are many models of collaboration, such as interprofessional collaboration, in this perspectives paper, we focus on transdisciplinary collaboration. We propose that transdisciplinary collaboration provides a greater opportunity for integrating allied health services in primary care, compared to multidisciplinary collaboration. We draw on examples of stroke rehabilitation to illustrate this argument. The following sections outline the context, define transdisciplinary collaboration, and explore potential benefits along with key implementation facilitators and barriers. We propose future directions for better integrating primary care for people with stroke.

## 2 Context: allied health professionals in primary care

*Australia’s healthcare system*, as the national context of the authors, is the focus of this paper. While we draw on some international evidence, it is not in the scope of this paper to provide comments on multiple healthcare systems. International readers should consider the perspectives presented within their own national context.

Allied health professionals, **in this paper**, refer to health practitioners who are not from the medical, nursing, or dental professions (5). However, there is no universal agreement on which professions are considered part of the allied health workforce. The United Kingdom’s National Health System lists 14 different professions, including art therapists, dietitians, orthoptists and radiographers (6). In Australia, the Department of Health, Disability and Ageing lists six example professions of physiotherapy, podiatry, psychology, pharmacy, occupational therapy, and social work (7). Allied Health Professions Australia provides seven criteria to define an allied health professional: (1) direct clinical care role, (2) associated with a national professional organization with defined codes and requirements, (3) accredited university course, (4) national entry level competency standards, (5) defined core scope of practice, (6) robust regulatory framework, and (7) use evidence-based practice (5). We have adopted the latter definition of an allied health professional in this paper.

*Primary care* refers to community-based health services that meet the four core characteristics outlined by the World Health Organization, being: (1) first contact, (2) continuous, (3) coordinated, and (4) comprehensive (8). Primary care is where individuals with non-emergency health concerns first seek help (8, 9), such as at their local general practice or an allied health clinic. Individuals then continuously receive healthcare by the same professional, team or organization, who coordinate the required services and referrals to address a comprehensive range of health needs. Those health needs may include prevention, diagnosis, treatment, and rehabilitation (8, 9). In this paper, we focus on private allied health clinics.

*Healthcare after stroke* may include referrals to healthcare and support services, secondary prevention, medication, and non-pharmacological interventions such as lifestyle change, dietary changes, and physical activity (10). In many countries, national stroke guidelines provide evidence-based recommendations for allied health professionals to enable community participation and long-term care (11). For example, in the Australian stroke guidelines, strong recommendations include self-management interventions (such as the Take Charge After Stroke program) and tailored carer support (11, 12). Other allied health interventions include escorted walking practice and adaptive equipment, targeted occupational therapy programs including leisure therapy, return to work assessment, and peer support groups (11). In this paper, we define primary stroke care as allied health-led assessment and/or interventions supported by stroke guidelines.

## 3 Models of collaboration

**Figure 1:**
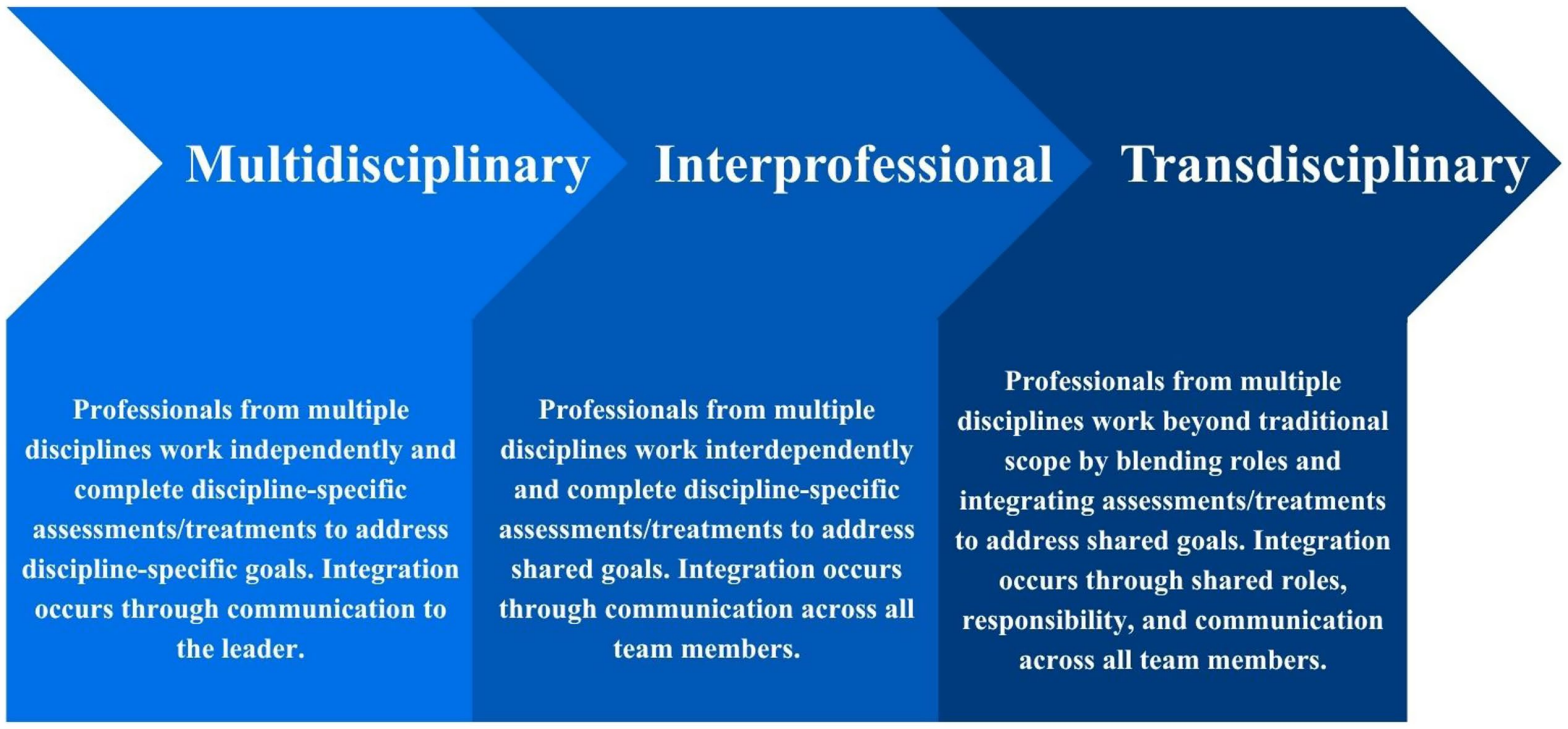
Example collaboration continuum for provision of integrated care (from multidisciplinary to transdisciplinary collaboration). Definitions adapted from Martin et al. (13).

There are many models of integrated care. While multidisciplinary and transdisciplinary collaboration are the focus of this perspectives paper, we recognize that these models sit on a continuum of collaboration. Therefore, in Figure 1, we have provided a visual of the collaboration continuum for the purpose of situating the reader (13–15). In the continuum, we have included multidisciplinary and transdisciplinary (focus of this paper) and interprofessional collaboration (an example of another collaboration model). For allied health professionals working in primary care, the most frequently described model of integrated care is multidisciplinary collaboration (4). When working within a multidisciplinary collaboration model for stroke care, allied health professionals typically deliver separate, discipline-specific assessments and/or treatments. Integration occurs only when results are communicated to a care coordinator (commonly the General Practitioner) (13–15) resulting in limited communication between health professionals (13, 14). This fragmented approach persists despite strong evidence that integrated primary care is important for individuals with chronic conditions, such as stroke, and whose complex needs extend beyond the expertise of a single discipline (1). Transdisciplinary collaboration provides a way to bridge this gap and deliver more integrated care.

Transdisciplinary collaboration provides a framework for professionals to share skills and safely transcend discipline boundaries through agreed scope extensions, clinical processes, and competency training (13, 14). Transdisciplinary collaboration models enable allied health professionals to take on roles and tasks in primary stroke care that are typically performed by another professional (4, 13). Integrating roles and negotiating discipline boundaries means that care is not segmented or divided based on the scope of each discipline (which occurs in multidisciplinary collaboration) (14). Rather, all team members communicate frequently and share equal responsibility for providing holistic care to address the individual’s needs (13, 14).

To illustrate the concept within our proposed context, we provide the following clinical example of allied health professionals providing multidisciplinary and transdisciplinary primary care for a person with stroke (see Box 1).

BOX 1 **Clinical example**

Judy experienced a stroke 6 months ago and was recently discharged from rehabilitation. She is still experiencing weakness and associated shoulder pain. The rehabilitation team referred Judy to a primary care clinic with a physiotherapist and an occupational therapist to continue her care.

*Multidisciplinary collaboration*: The physiotherapist completed a strength assessment and gait assessment before prescribing leg strengthening exercises and a graded endurance program. The physiotherapist did not review Judy’s arm strength, as this is the role of the occupational therapist at the clinic. The occupational therapist completed a strength assessment and task analysis before prescribing functional upper limb exercises and recommending positioning to minimize pain and support successful task performance. However, the occupational therapist did not prescribe a sling to support the shoulder joint to reduce pain, as sling prescription is the role of the physiotherapist at the clinic. The physiotherapist and occupational therapist provided separate reports on their assessment and interventions to Judy’s General Practitioner. While Judy felt the clinical care she received was of high quality, she thought the services were disconnected as she completed some assessments twice, was prescribed two sets of exercises, was given different recommendations on how frequently to complete the exercises, and needed to coordinate appointments with two disciplines. She also experienced gaps in care, her shoulder pain was not holistically addressed, as the clinicians assumed the other would assess or prescribe a sling.

*Transdisciplinary collaboration*: The occupational therapist and physiotherapist discussed the referral before the physiotherapist, who had previously completed additional upper limb stroke rehabilitation training to work in an expanded scope of practice role, accepted the referral. The physiotherapist completed a strength assessment, task analysis and gait assessment before recommending positioning to support successful task performance and prescribing functional strengthening exercises and a graded endurance program. The physiotherapist prescribed a sling to support the shoulder joint to reduce pain. The physiotherapist shared the assessment results and treatment plan with the occupational therapist to verify a specialist occupational therapy referral was not required. The physiotherapist then provided a report on the assessment and interventions to Judy’s General Practitioner. The communication between the physiotherapist and occupational therapist prevented (1) professional boundary conflicts, which could have occurred because the physiotherapist completed tasks that might typically fall within the scope of the occupational therapist, and (2) gaps in care if a professional’s scope of practice is constrained. Judy thought the clinical care was comprehensive and holistically addressed her goals. Judy completed the assessments once, was prescribed one exercise regimen, and needed to coordinate appointments with only one discipline.

## 4 Opportunities and benefits

To our knowledge, no studies on allied health transdisciplinary collaboration models have been carried out in primary care settings, such as private allied health clinics. We therefore draw on evidence from transdisciplinary collaborations from other settings (e.g., hospitals) to illustrate potential benefits and highlight why transdisciplinary collaboration is a logical approach for allied health professionals providing stroke care in primary care settings.

### 4.1 Opportunity: service gaps in long-term care for people after stroke

Some people of working age with stroke report low mood, poor quality of life and failure to return to work after 12 months (16). Long-term and integrated stroke rehabilitation (i.e., care provided by specialist teams outside of dedicated inpatient and outpatient centers) is not readily available (17). For example, in an Australian study, only a small proportion of people with stroke accessed rehabilitation at home (11%) and at primary care clinics (2%) (18). In other studies, a large proportion of younger people (18–45 years) and those who experienced a mild stroke did not receive rehabilitation (30–47% and 34–38%, respectively) (16), and the psycho-emotional and cognitive needs for younger people were not addressed (19). The service gap persists as stroke rehabilitation is designed around funding mechanisms and policies which mandate access at specific times and specific places. One consumer described their experience thus, “I was still in denial about this actually happening to me, [so did not take up what was offered] … I think once you’re out of denial and actually go “Okay, this has happened, and now what am I going to do about it?”, *that’s when you need to be able to access rehab*” (17). Given the evidence of unaddressed needs, there is an opportunity for allied health professionals in primary care to provide integrated long-term stroke rehabilitation.

### 4.2 Benefit: embracing overlapping skills, reducing duplication

Allied health professionals possess overlapping skills, often resulting in duplicated effort (20, 21). The areas in which allied health professionals overlap are a natural starting point for transdisciplinary collaboration. For example, physiotherapists, podiatrists and audiologists possess overlapping skills in balance assessment. Occupational therapists and speech pathologists overlap in assessment of cognitive-communication impairments, such as information processing and memory (22, 23). In hospital settings, transdisciplinary collaboration has been shown to reduce duplication of clinical tasks, resulting in better use of staff time and skills (23) and improved patient experience (24). When the transdisciplinary collaboration model was not used, one participant stated that assessment “*wastes a lot of time repeating some activities and questions*” (24). Therefore, transdisciplinary collaboration models that are based on skills that are already shared across professions could be well-received (and less likely to be resisted) by allied health professionals and clients in primary care.

### 4.3 Opportunity: supply–demand crisis

Primary care, like all healthcare contexts, faces a global supply and demand crisis (25). The crisis is due to the compounding effect of multiple factors—global workforce shortages, aging populations with higher prevalence of chronic conditions, and the persistence of siloed, multidisciplinary models of collaboration (9, 21, 25). Addressing this crisis requires a multifaceted approach, with better integration of care likely to be critical. Studies have shown that integrating how professionals operate in primary care reduces waiting times and service duplication, resulting in more time and cost-efficient utilization of healthcare services (1, 4, 26). In an Australian acute stroke unit, a transdisciplinary collaboration model reduced allied health professional time spent on assessment by 37.6 min per patient (95% CI −47.5, −27.7, *p* < 0.001), reduced the median occasions of service required (*p* = 0.011), and saved an estimated AU$379.45/admission (27). While the potential organizational and societal cost savings in primary care settings are unknown, the estimated average out-of-pocket expense for people after stroke is AU$386.4/year (28). The authors expect that workforce and service efficiency benefits could be achieved for primary stroke care if a transdisciplinary collaboration was adopted.

### 4.4 Benefit: client and staff outcomes

Integrating care provision in primary care settings improves chronic disease management, health outcomes, healthcare access and satisfaction with care (1, 4, 26). For providers, integrated care improves performance, clinical decisions (e.g., appropriate referrals), work satisfaction, and reduces burnout (1, 4, 26). Although evidence of transdisciplinary collaboration models in allied health primary care clinics is not available, hospital-based studies report high levels of client and staff satisfaction with transdisciplinary collaboration across multiple groups, including people with acute stroke (23, 24). We can draw on evidence from community-based studies. In a first contact physiotherapy musculoskeletal clinic, clients appreciated streamlined services that created earlier access to intervention, for example, “*I didn’t need to explain everything twice. The physio knew what to ask and got straight to it*” (29). In a transdisciplinary home-visiting community service, skill sharing between occupational therapists and physiotherapists maintained mobility, function, and quality of life outcomes for older adults (21). Similar benefits for staff and clients could be achieved if transdisciplinary collaboration models are implemented in primary care.

### 4.5 Opportunity: scalability and impact

The authors propose that the potential for impact of transdisciplinary collaboration is significant due to the widespread siloed approach to primary care delivery (4). These silos exist and persist due to culture, organizational and funding structures (4, 25, 26), which we will discuss later in detail. There is opportunity for system-level reform to reorganize healthcare delivery and reimbursement. This could be done in a way that encourages workforce integration using transdisciplinary collaboration to address complex health issues such as stroke (9). Potential benefits of transdisciplinary collaborations (such as time and cost savings, as discussed earlier) could be realized across primary care settings.

## 5 Facilitators and barriers

While there are potential benefits for allied health professionals using transdisciplinary collaborations in primary care, it is important to consider implementation feasibility and sustainment. In the absence of direct evidence, we draw on facilitators and barriers identified in the wider transdisciplinary and integrated care literature, and explore these at individual, team, organizational, and societal levels.

### 5.1 Individual level

At an individual level, transdisciplinary collaboration models are successful when the professionals involved are willing to share their skills, knowledge and roles (23). When roles are shared, professionals must trust each other and perceive each other as competent to safely complete the roles (23). If individuals do not trust each other, lack confidence in one another’s skills, or lack competence themselves, then transdisciplinary collaboration can fail (24). For example, if an occupational therapist and speech pathologist share the responsibility of cognitive assessments, but lack confidence in each other’s competence, they are likely to revert to siloed practice. In this scenario, structured opportunities to observe each other, provide feedback, and address concerns are essential for rebuilding trust and supporting true collaboration.

### 5.2 Team level

At a team level, collaboration can be hindered by inadequate team composition, the presence of hierarchy, and poor team culture and leadership (2, 4, 26, 30). For transdisciplinary collaboration, team size and skill mix (including competency and practice scope of individuals) should match consumer needs and demand. Importantly, all team members must contribute as equals without financial or power hierarchies. Leaders can implement flat team structures or promote a non-hierarchical culture to facilitate equity, shared decision-making, and openness to diverse perspectives, all of which are essential for building a trusting and collaborative team culture (26). Without these elements, attempts at transdisciplinary collaboration are likely to be undermined by inadequate team capabilities, inequity, and team member resistance.

Another team-level consideration for transdisciplinary collaboration is structure and clinical governance. The team and its function need to be well-defined, including clearly defined roles, standardized care protocols (e.g., stroke guidelines), and learning/supervision pathways (e.g., competency pathways) (23–26, 30). For transdisciplinary collaboration, it is essential to define who is doing what, when, and how (24). Without such clarity, health professionals may be underutilized (i.e., choose to stick to old ways of working), inadvertently provide care for which they are not qualified, challenge and resist the notion of refining scope boundaries, or provide duplicated services.

### 5.3 Organizational level

At an organizational level, unreasonable performance expectations, high workloads, and ethical and legal considerations can be barriers to collaboration (20, 25, 30). Unreasonable performance expectations and high workloads leave clinicians time-poor. These factors could result in limited time to learn new transdisciplinary skills, become familiar with transdisciplinary protocols, complete essential competency training, discuss client cases with colleagues, or engage in supervision. However, from a business perspective, time for collaboration does not directly generate revenue and could be difficult to justify. Implementation and sustainment of a new transdisciplinary collaboration model requires dedicated time for planning, training and collaboration, facilitated by flexible funding structures.

Ethical and legal concerns can also arise when multiple professionals are involved in patient care and shared accountability (30). In transdisciplinary collaboration, where traditional scope boundaries are redefined and clinicians share care provision responsibility, issues of accountability and associated concerns about liability are likely. For example, who is responsible for providing care if more than one professional is skilled? And what are the medicolegal ramifications for completing a task beyond the usual scope of practice? Moreover, clinicians may be concerned about funding implications if they do not complete a clinical assessment/task themselves. For example, the National Disability Insurance Scheme (NDIS) in Australia requires extensive assessment by a professional before the provision of clinical care by that professional. Such barriers could be addressed if: effective communication pathways are established between practitioners; funding rules in relation to transdisciplinary collaboration are understood; and transdisciplinary protocols (including competency and liability-sharing frameworks) are co-designed (30). Addressing these policy, regulatory, and funding considerations is essential to ensure transdisciplinary collaboration models can be implemented safely, legally and sustainably.

### 5.4 Societal level

At a societal level, the main feasibility considerations pertain to policy, regulation and funding. Transdisciplinary collaborations must be considered alongside professional practice guidelines and regulatory restrictions on what allied health professionals can do (4, 20, 25, 31). For example, are occupational therapists supported by practice guidelines to deliver community mobility interventions for people with stroke instead of (or in addition to) physiotherapists? Are there legal restrictions for physiotherapists who deliver nutrition education for stroke prevention instead of (or in addition to) dieticians? Are there additional legal requirements (such as competency pathways and supervision) for practice scope extension?

In addition to policy, there are funding considerations that may affect the implementation of transdisciplinary collaboration in primary care. It is well-documented that compensable funding models (e.g., fee-for-service) limit collaboration, as collaboration is unbillable work (4, 26). Other funding models, such as salaries rather than contracting arrangements, provide secure and consistent payment, which may facilitate collaboration (4, 26). Transdisciplinary collaboration has the potential to operate under both funding models, provided the funding and care models are aligned. For example, if an allied health professional is highly skilled, has completed extensive additional training, and is competent to provide services on behalf of other disciplines, then a fee-for-service model may be appropriate. Alternatively, if multiple allied health professionals collaborated on a complex case, a salary funding model may be more suitable. However, in some countries, adapting a salary-funded model is challenging. For example, Australian private practices largely operate on a fee-for-service basis, where Medicare and private health insurance payments are tied to discipline-specific item numbers. This structure does not reimburse or incentivize the time spent on collaboration or shared care planning between allied health professionals. This situation creates a financial disincentive for transdisciplinary collaboration. In addition, while most allied health professionals in primary care receive a base salary, they may also receive bonuses depending on the number of clients seen. This scenario means that allied health professionals are incentivized to see more clients, and time spent undertaking collaboration activities would negatively impact earning potential. Without state and national health reform to include funding mechanisms that recognize and reward integrated care, the feasibility of transdisciplinary collaboration in private practice will remain limited.

## 6 Discussion

Transdisciplinary collaboration between allied health professionals has a role in primary care that is worthy of further investigation. In this paper, we discussed primary stroke care. We outlined potential benefits of transdisciplinary collaboration, including reduced service duplication, more efficient and cost-effective services, and client and staff outcomes. We also discussed potential facilitators and barriers to transdisciplinary collaboration (such as the health practitioner’s competence, team composition and culture, clinical governance, workload, ethical and regulatory guidelines, and existing funding models).

The next step is to determine how transdisciplinary collaboration should be implemented within primary care. Throughout this paper, we provided clinical examples of primary stroke care. The examples illustrate how allied health professionals can share skills and redefine their practice scope to provide more streamlined stroke care. The authors do not suggest that all allied health professionals who provide stroke care in primary care settings should use transdisciplinary collaborations. Instead, the appropriate model should be selected from the collaboration continuum (Figure 1).

Appropriate selection of a collaboration model is important for successful and optimal care integration (23). While this perspectives paper focuses on multidisciplinary and transdisciplinary collaboration, we recognize that other models that we have not discussed (e.g., interprofessional collaboration) could be more appropriate in some settings. Therefore, it is essential to use a framework to identify if a transdisciplinary collaboration model is appropriate. Previous work has outlined four considerations when selecting a multidisciplinary, interprofessional, or transdisciplinary collaboration model. The first consideration is the “healthcare setting and jurisdiction,” which encompasses the outer context and external factors including the service/clinic, internal policies, professional practice guidelines, regulatory restrictions, and funding models (32). The second consideration is “consumer needs,” including the healthcare requirements and goals of the consumer group (32). The third consideration, “workforce available,” refers to the healthcare professionals and workforce factors such as staff turnover, team composition, and team culture (32). The fourth consideration is “clinician attributes and skills”, comprising an individual’s knowledge, skills, values, motivations and preferences (32). Based on our analysis of potential facilitators and barriers, we propose one additional, novel consideration, “team collaboration rules”. This fifth consideration encompasses the leadership, communication and collaboration structures/protocols, and clinical governance requirements to foster collaboration. An example of “team collaboration rules” for multidisciplinary and transdisciplinary collaboration models is provided in Table 1.

**Table 1:** Clinical example of the five considerations for the selection of a multidisciplinary or transdisciplinary collaboration model.

Considerations	Multidisciplinary collaboration	Transdisciplinary collaboration
Clinical scenario	A client experienced a stroke 1.5 years ago and is working with allied health professionals to improve his gait, balance, ankle position, and tone.	
Consideration 1: healthcare setting and jurisdiction (determined by external factors)	Physiotherapy, podiatry, and massage therapy are located in the same clinic and use a fee-for-service model.	Physiotherapy, podiatry, and massage therapy are located within the same clinic and use a fee-for-service model.
Consideration 2: consumer needs	A long-term goal is to improve gait. The client will require multiple physiotherapist and podiatrist appointments. The client has the time and capacity to attend multiple appointments with different clinicians each month.	A long-term goal is to improve gait. The client will require multiple physiotherapist and podiatrist appointments. The client has the time and capacity to attend one appointment each month and likes to see the same clinician, therefore prefers to book with the physiotherapist who is also skilled to identify footwear that will provide the best support.
Consideration 3: workforce available	The clinicians provide specialized services, work within a defined scope of practice, participate in professional development directly related to their practice scope, and communicate recommendations to the patient’s General Practitioner. There is no funded time to participate in team meetings, there is only time for the clinicians to provide services within a strict scope of practice.	The clinicians have good interpersonal relationships. As a result, clinicians have developed an in-depth understanding of each other’s roles, areas of overlap, and engage in professional development to share skills. While there is no funded time to participate in multidisciplinary team meetings, the clinicians save time by working within an approved expanded scope of practice and identifying specific, top-of-scope referrals to other disciplines. The clinicians use the time saved to participate in monthly case conferences as a source of regular professional development by discussing their complex cases with other discipline experts.
Consideration 4: clinician attributes and skills	The physiotherapist is a new graduate with foundation knowledge, limiting the ability to identify referrals to other disciplines. The podiatrist and massage therapist believe that providing highly specialized services is the best way to address client goals.	The physiotherapist has completed additional training to assess foot position. The podiatrist trusts the physiotherapist to complete this assessment. Both clinicians understand the efficiency and client-centered benefits of sharing skills.
Consideration 5: team collaboration rules	Time to communicate with and refer to clinicians at other clinics is not financially compensated or encouraged. However, the clinicians are required to provide clinical summaries to the patient’s General Practitioner, who may or may not refer to other clinicians.	Clinicians are paid a salary, allowing flexibility to schedule case conferences and complete appointments on behalf of another discipline. Because of the ability to collaborate, a primary clinician is identified to coordinate patient care across disciplines and is responsible for seeking advice from or coordinating sessions with other clinicians when the patient’s goals exceed their own expertise.

Healthcare teams should investigate and understand all five considerations before selecting a collaboration model. That is, the local context will impact on which collaboration model is the most appropriate in any given setting. If one variable changes (e.g., consumer cohort or funding model), then the best collaboration model could also change. To illustrate the reasoning required, we have provided a clinical example and applied the five considerations to multidisciplinary and transdisciplinary collaboration models in Table 1.

### 6.1 Limitations

This paper has two main limitations. First, the lack of direct evidence on transdisciplinary collaboration models in primary care. As a perspectives paper, we draw on related evidence fields and professional experience to help inform future implementation and clinical research. Second, the facilitators and barriers will vary across individuals, teams, organizations and nations. This paper draws on the Australian context, and therefore, generalizability of the perspectives to other jurisdictions and nations could be limited.

### 6.2 Future directions

#### 6.2.1 Implementation of transdisciplinary collaboration models

There is a need for transdisciplinary collaboration implementation and research within primary care and between nations. Implementation of transdisciplinary collaboration models could result in provision of more efficient care and improved patient outcomes. Therefore, we challenge allied health professionals, leaders and managers in primary care, as well as policy makers, to review current practice and explore avenues for transdisciplinary collaboration for primary stroke care. The stakeholders should take the following actions:

1. Use the five considerations (healthcare settings and jurisdiction, consumer needs, workforce available, clinician attributes and skills, and team collaboration rules) as a framework to decide if transdisciplinary collaboration is the most suitable model.
2. Identify professional skills that are duplicated and/or suitable for skill-sharing.
3. Create a clinical process based on the stroke guidelines and professional skills.
4. Use the five considerations to identify potential individual, team, organizational and societal facilitators and barriers.
5. Select implementation strategies and plans to address barriers.

There could be value in implementing transdisciplinary collaboration models more broadly in primary care. For example, the actions listed above could apply to other chronic conditions. Further consideration and research are required.

#### 6.2.2 Policy and funding considerations

For implementation to be successful, one critical barrier that would need to be addressed is available funding models – decision-makers and health policy leaders need to advocate for funding solutions that will incentivize collaboration. This perspectives paper focused on private allied health clinics. However, the learnings should also be considered in other settings that were beyond the scope of this paper. For example, future papers should consider policy and funding solutions that will incentivize collaboration in settings where post-stroke care is provided by social and community professionals, such as within community and home care organizations. Analysis of the issues associated with siloed post-stroke healthcare in these settings is a logical next step. Stakeholders should report and publish implementation efforts and results to propel primary care beyond siloed multidisciplinary collaboration models.

## Data Availability

The original contributions presented in the study are included in the article/supplementary material, further enquiries can be directed to the corresponding author.
